# Interactions between Naïve and Infected Macrophages Reduce *Mycobacterium tuberculosis* Viability

**DOI:** 10.1371/journal.pone.0027972

**Published:** 2011-11-18

**Authors:** Michelle L. Hartman, Hardy Kornfeld

**Affiliations:** Department of Medicine, University of Massachusetts Medical School, Worcester, Massachusetts, United States of America; Fundació Institut d'Investigació en Ciències de la Salut Germans Trias i Pujol, Universitat Autònoma de Barcelona, CIBERES, Spain

## Abstract

A high intracellular bacillary load of *Mycobacterium tuberculosis* in macrophages induces an atypical lysosomal cell death with early features of apoptosis that progress to necrosis within hours. Unlike classical apoptosis, this cell death mode does not appear to diminish *M. tuberculosis* viability. We previously reported that culturing heavily infected macrophages with naïve macrophages produced an antimicrobial effect, but only if naïve macrophages were added during the pre-necrotic phase of *M. tuberculosis*-induced cell death. In the present study we investigated the mechanism of antimicrobial activity in co-cultures, anticipating that efferocytosis of bacilli in apoptotic bodies would be required. Confocal microscopy revealed frustrated phagocytosis of *M. tuberculosis*-infected macrophages with no evidence that significant numbers of bacilli were transferred to the naïve macrophages. The antimicrobial effect of naïve macrophages was retained when they were separated from infected macrophages in transwells, and conditioned co-culture supernatants transferred antimicrobial activity to cultures of infected macrophages alone. Antimicrobial activity in macrophage co-cultures was abrogated when the naïve population was deficient in IL-1 receptor or when the infected population was deficient in inducible nitric oxide synthase. The participation of nitric oxide suggested a conventional antimicrobial mechanism requiring delivery of bacilli to a late endosomal compartment. Using macrophages expressing GFP-LC3 we observed the induction of autophagy specifically by a high intracellular load of *M. tuberculosis*. Bacilli were identified in LC3-positive compartments and LC3-positive compartments were confirmed to be acidified and LAMP1 positive. Thus, the antimicrobial effect of naïve macrophages acting on *M. tuberculosis* in heavily-infected macrophages is contact-independent. Interleukin-1 provides an afferent signal that induces an as yet unidentified small molecule which promotes nitric oxide-dependent antimicrobial activity against bacilli in autolysosomes of heavily infected macrophages. This cooperative, innate antimicrobial interaction may limit the maximal growth rate of *M. tuberculosis* prior to the expression of adaptive immunity in pulmonary tuberculosis.

## Introduction

A role for apoptosis in innate defense against *Mycobacterium tuberculosis* (*M.tb*) was suggested by evidence that attenuated *M.tb* H37Ra and *M. bovis* BCG induced tumor necrosis factor (TNF)α stimulated apoptosis in infected macrophages, which was associated with a reduction in bacillary viability [1], [2]. In addition to the direct antimicrobial processes occurring in apoptotic macrophages, a previous study by Fratazzi et al. [3] demonstrated that the addition of naïve macrophages to macrophages infected with an apoptosis-inducing strain of *M. avium* strongly inhibited bacillary growth. This co-culture effect was observed when the infected cells were apoptotic, but not if they were made necrotic by sonication.

The host-protective effects of apoptosis in tuberculosis (TB) is now an accepted paradigm but its biological relevance is uncertain since virulent *M.tb* inhibits the apoptotic death of infected macrophages [2] and uses these cells as a replication niche. Lee et al. [4] reported that virulent *M.tb* induces an atypical, ultimately necrotic mode of macrophage cell death at threshold intracellular burden of 25 bacilli per macrophage. In the first several hours after high multiplicity of infection (MOI) challenge, infected macrophages have apoptotic features of nuclear condensation and phosphatidylserine (PS) externalization without apoptotic vesicle formation. Adding naïve macrophages during this pre-necrotic interval was shown to inhibit *M.tb* replication, whereas adding naïve macrophages at 24 h post-infection when the infected population was necrotic had no antimicrobial effect.

In the present study we investigated the mechanism responsible for inhibiting *M.tb* replication in co-cultures of naïve and infected macrophages, initially testing the hypothesis that this depended on phagocytosis of apoptotic bodies (efferocytosis) by the naïve population. Results did not support that hypothesis: confocal microscopy demonstrated incomplete engulfment of infected macrophages (“frustrated efferocytosis”) and the antimicrobial effect contributed by naïve macrophages was found to be contact-independent. We show that naïve macrophages are stimulated in an interleukin-1 receptor (IL-1R)-dependent manner to produce a soluble factor that acts back on heavily infected macrophages to restrict *M.tb* growth in a nitric oxide-dependent manner. This implies the delivery of bacilli to a late endosomal compartment, and in that regard we showed that a high intracellular *M.tb* load is sufficient to induce autophagy in macrophages. Our data reveal crosstalk between infected and uninfected macrophages that inhibit *M.tb* replication, most likely by promoting antimicrobial processes in autolysosomes prior to the completion of *M.tb*-induced necrosis. These interactions may serve to limit the maximal proliferative potential of *M.tb* during the early phase of pulmonary TB before the expression of adaptive immunity.

## Results

### Cell contact is not required for co-culture antimycobacterial activity

**Figure 1:**
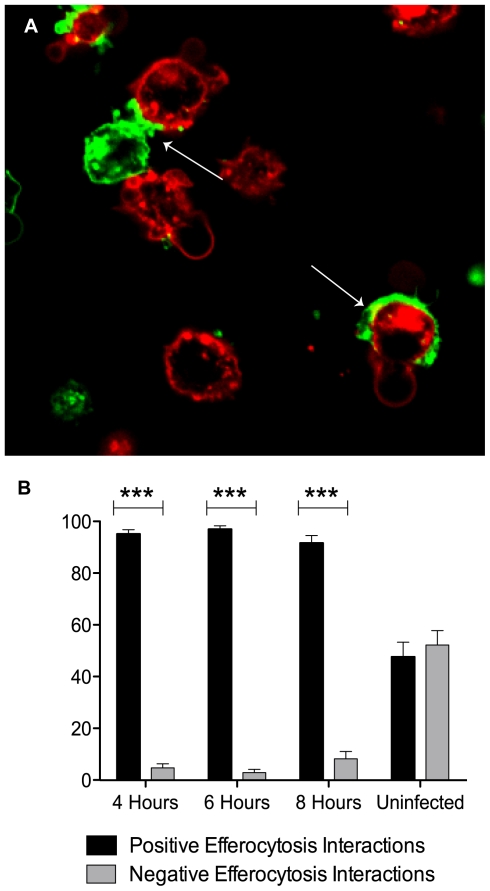
Attempted engulfment of *M.tb*-infected macrophages by naïve macrophages is incomplete and unidirectional. (**A**) Infected macrophages (*red*) were co-cultured with uninfected macrophages (*green*) for 3 h. Samples were fixed and observed by laser-scanning confocal microscopy. White arrow identifies early contact points where naïve macrophages form pedestals or partially engulf infected macrophages. (**B**) Directionality of frustrated efferocytosis was quantified by counting the number of “positive” interactions, ones in which naïve macrophages form pedestals or partially engulf infected macrophages, compared to “negative” interactions where cell-cell contact occurred without pedestals or engulfment (all interactions not described as “positive”). Results are expressed as the % positive interactions vs. % negative interactions at each time point ± SD. ***, *p*<0.0001 for all time points.

Previous studies of macrophages infected with *M. avium* or *M.tb* demonstrated that co-culture with naïve macrophages restricted mycobacterial growth, but only when the originally infected cells exhibited features of apoptosis [3], [4]. It was speculated in those reports that antimycobacterial activity might depend on uptake of bacilli by uninfected macrophages via efferocytosis of apoptotic host cells, but this was not formally tested. To investigate that question we first assessed whether efferocytosis occurs in co-cultures of naïve cell line macrophages stained with Cholera toxin 488 and *M.tb* Erdman-infected macrophages (MOI 50) stained with Cholera toxin 647. Under these conditions the infected population is committed to death and externalizes PS but would not have progressed to necrosis, which is not widespread until 24 h post-infection. Samples were fixed at 4, 6, and 8 h after co-culture and examined by confocal microscopy. Interactions between infected and uninfected macrophages consistent with early or attempted engulfment were evident at all time points. Figure 1A shows pedestal formation and partial engulfment of whole infected cells at the synapse between these cell populations (white arrows). Complete internalization of infected macrophages was not observed even after 24 h of co-culture. These interactions were almost exclusively unidirectional, with uninfected cells forming pedestals on infected macrophages or partially engulfing them (Fig. 1B). This indicated that these interactions were not random but mediated through an “eat me” signal such as PS on the surface of the infected cells. We did not observe apoptotic vesicle formation in the infected macrophage population, consistent with the morphology of cells undergoing high MOI *M.tb*-induced cytolysis we previously described [4]. Nonetheless, it is formally possibly that vesicles were formed at a low rate and were either ingested by naïve macrophages or degraded by secondary necrosis. Our failure to see any evidence for this phenomenon suggests that if it occurred at all it did not play a major role in the conditions of our experiments. The apparent failure to completely internalize all or parts of infected macrophages suggests that these interactions (in the absence of apoptotic vesicle formation) might reflect frustrated efferocytosis by analogy to frustrated phagocytosis [5].

**Figure 2:**
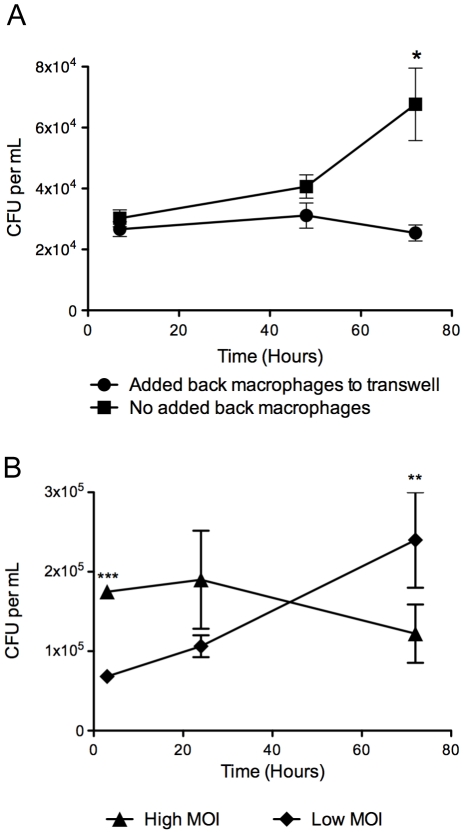
Interactions between infected and naïve macrophages results in the production of a soluble factor that reduces M.tb viability. (**A**) Macrophages infected with *M.tb* Erdman (MOI 50) were cultured alone or in the presence of uninfected macrophages separated by transwell polycarbonate membranes. Results are expressed as mean CFU per mL ± SD at the indicated time points. *, p = 0.018 comparing co-cultures of *M.tb*-infected plus uninfected macrophages to infected macrophages alone at 72 h. (**B**) Filter-sterilized supernatant from macrophage co-cultures was added to wells contained isolated, infected macrophages alone. Transfer of co-culture supernatant reduced *M.tb* viability demonstrating that the factor responsible for the effect is soluble. *, *p*<0.0001; **, *p* = 0.002.

To test whether physical contact between naïve and infected macrophages was required to limit *M.tb* replication, infected and uninfected macrophages were separated with a 0.4 µm pore size polycarbonate transwell filters for the duration of the co-culture. CFU was measured at 3, 48 and 72 h post-addition of naïve macrophages. As shown in Figure 2A, separation of the macrophage populations by transwell did not change the outcome of the co-culture antimycobacterial effect. This demonstrated that contact between naïve and *M.tb*-infected macrophages is not necessary for this phenomenon to occur, implying the production of a soluble factor by naïve macrophages acting to limit *M.tb* replication in during the early, pre-necrotic phase of cell death in the infected population.

To test whether a soluble factor expressed in co-cultures was sufficient for the observed antimycobacterial effect, we took supernatants from cell line co-cultures of naïve and *M.tb*-infected macrophages 3 h after co-culture began. Supernatants were filter-sterilized (0.22 µm) to remove bacilli or debris before being added to new cultures of infected cell line macrophages and then CFU was measured 3, 24 and 72 h later. Antimicrobial activity was transferrable (Fig. 2B) and operated only in the context of infected macrophages in the early, apoptotic, phase of *M.tb*-induced death. These results indicated that the co-culture antimycobacterial effect was mediated by a soluble factor produced by naïve macrophages that acted on infected, pre-necrotic macrophages to limit *M.tb* growth. This activity was produced by naïve macrophages within 3 h of co-culture and did not require physical contact with infected macrophages, implying also the existence of a soluble afferent factor produced by the dying cells.

### Interleukin-1 is an afferent signal in co-cultures

**Figure 3:**
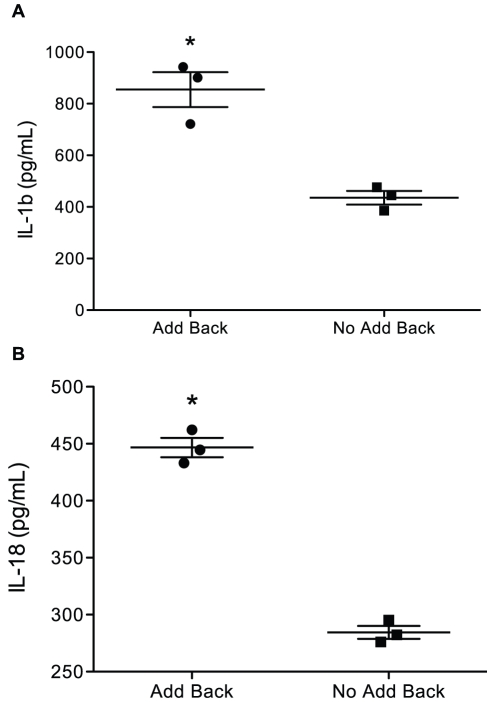
Cytokine profile of *M.tb-*infected macrophages in the presence or absence of naïve macrophages. Macrophages were infected with *M.tb* Erdman (MOI 50) for 3 h then cultures were washed and the infected cells were resuspended in medium alone or medium plus an equal number of naïve macrophages. After an additional 3 h in culture, supernatants were collected, and frozen at −80°C until multiplex cytokine analysis. Concentrations of IL-1β and IL-18 are shown for co-cultures of infected and uninfected macrophages (*add back*) or culture of *M.tb*-infected macrophages without naïve macrophages (*no add back*).

**Figure 4:**
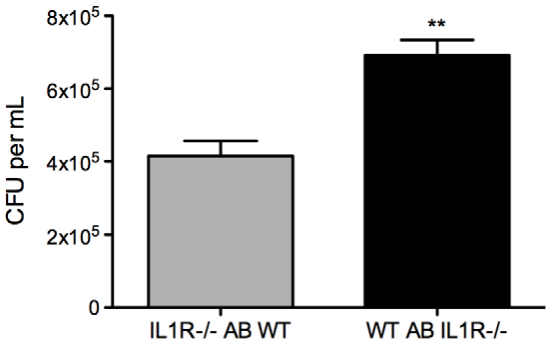
Antimycobacterial activity in macrophage co-cultures requires IL-1R expression on the naïve cell population. Co-cultures were established with *M.tb*-infected IL-1R^−/−^ macrophages and uninfected wildtype macrophages (*IL-1R^−/−^ AB WT*) or vice versa (*WT AB IL-1R^−/−^*). CFU was measured at 0 and 48 h post-addition of naïve macrophages. Results are expressed as the difference in mean CFU/ml ± SD from time zero to 48 h. **, *p* = 0.009.

The involvement of soluble signals in the crosstalk between infected and uninfected macrophages in our system suggested a possible role for cytokines. A screen of candidates by ELISA of co-culture supernatants demonstrated expression of several cytokines by infected macrophages in the absence of co-culture, most of which further increased upon the addition of naïve macrophages (Fig. 3). Among these were IL-1β, and IL-18. Based on that finding, we tested whether IL-1R signals were required as an afferent, death-associated signal to induce production of a secreted efferent factor from naïve macrophages, or to mediate the efferent effect of limiting *M.tb* replication in infected macrophages. Co-cultures were established, using IL-1R^−/−^ cell line macrophages to replace wildtype cells in either the infected or naïve population (Fig. 4). These experiments demonstrated that the co-culture antimycobacterial activity required IL-1R expression on naïve but not on infected macrophages in this system. This suggested that IL-1β served as an afferent signal to directly or indirectly induce the expression of an unidentified soluble efferent factor by naïve macrophages in the co-culture system.

### The efferent signal in co-cultures is elusive

Having found at that IL-1β participates as an afferent factor in the co-culture system we sought to understand the mechanism of its expression and also to identify the factor present in co-culture supernatant that inhibited *M.tb* replication in the infected macrophages. It was previously suggested that P2X_7_ receptor (P2X_7_ R) signaling, activated by ATP can activate the NLRP3 inflammasome leading to caspase-1 activation and cleavage of pro-IL-1 resulting in IL-1β release [6], [7] Moreover, stimulation of P2X_7_R in *M.tb*-infected macrophages was shown to induce antimycobacterial activity by directing bacilli to autophagosomes [8], [9]. We investigated the involvement of ATP-mediated signaling by establishing co-cultures where either the infected or the uninfected primary macrophages were derived from bone marrow of P2X_7_R^−/−^ mice. Regardless of what population was deficient of P2X_7_R, there was no reduction of antimycobacterial activity (Fig. S1a). This result indicated that P2X_7_R signaling was not required either for afferent or efferent limbs of antimicrobial activity observed in the co-culture system.

Adenosine is another nucleotide with immunomodulatory effects that is released from stressed and dying cells. It was reported that IL-1β increases adenosine receptor expression on monocytes and increases responsiveness to adenosine [10]. In co-cultures established with primary macrophages from bone marrow of adenosine A2a receptor^−/−^ mice we found no reduction of antimycobacterial activity when these cells were used either as the naïve or infected population (Fig. S1b). Furthermore, treating infected macrophages with the stable adenosine agonist NECA did not limit *M.tb* replication (data not shown). These results effectively excluded a requirement for adenosine in the co-culture system.

To define the physical nature of the efferent co-culture factor, supernatants were treated with DNAse, RNAse, proteinase K and boiling (Fig. S2). None of these treatments reduced the antimycobacterial effect conferred by co-culture supernatants. Passing co-culture supernatant through a series of molecular size exclusion filters to a minimum of 3 kDa also failed to deplete the activity. Treating naïve cell line macrophages with indomethacin also failed to diminish the activity, indicating the efferent factor or its production did not depend on cyclooxygenase enzymes in the naive cells. These experiments revealed that the efferent factor in this system is a heat-stable small molecule and not likely to be a protein, nucleic acid, prostaglandin or thromboxane. The identity of this factor remains to be determined.

### Mechanism of antimycobacterial activity in co-cultures

**Figure 5:**
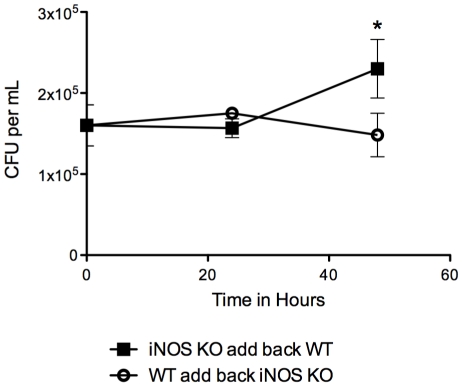
Antimycobacterial activity in macrophage co-cultures requires iNOS expression in the infected cell population. Co-cultures were established with *M.tb*-infected iNOS^−/−^ macrophages and uninfected wildtype macrophages (*iNOS−/− AB WT*) or vice versa (*WT AB iNOS−/−*). CFU was measured at 0, 24 and 48 h post-addition of naïve macrophages. Results are expressed as mean CFU/ml ± SD at the indicated times. *, *p*<0.01 comparing CFU between groups at 48 h.

Having failed to identify the soluble efferent factor in the co-culture experiments we turned our attention to the cellular requirements for the observed antimicrobial effect. Our previous experiments indicated that the factor acted only on dying macrophages before the onset of necrosis, which suggested that the efferent factor induced or facilitated an intracellular antimicrobial process in the infected macrophages. Nitric oxide is a major factor limiting *M.tb* growth in macrophages; a function that has been demonstrated in vitro and in vivo [11]–[14]. To investigate any involvement of nitric oxide in the antimycobacterial effects observed in our experiments, we established co-cultures using inducible nitric oxide synthase (iNOS)^−/−^ cell line macrophages as the infected or the naïve population (Fig. 5). When iNOS^−/−^ macrophages were the infected cell population, co-culture with naïve macrophages failed to limit *M.tb* growth. In contrast, naïve iNOS^−/−^ macrophages were capable of stimulating antimycobacterial activity in wildtype macrophages infected with *M.tb*. This result suggested that the efferent factor released from naïve macrophages facilitated a conventional antimycobacterial function in the infected cells that acts on bacilli in mature phagolysosomes. A cardinal feature of *M.tb* virulence is the capacity to arrest phagosome maturation and establish an intracellular compartment resembling an early endosome that is permissive for bacillary replication [15]. In this regard, Davis et al. [16] reported that iNOS is excluded from *M.tb* phagosomes. Results from our experiment with iNOS^−/−^ macrophages implied that in heavily infected macrophages, bacilli may be located in acidified lysosomal compartments.

### *M.tb* infection directly induces autophagy in macrophages

**Figure 6:**
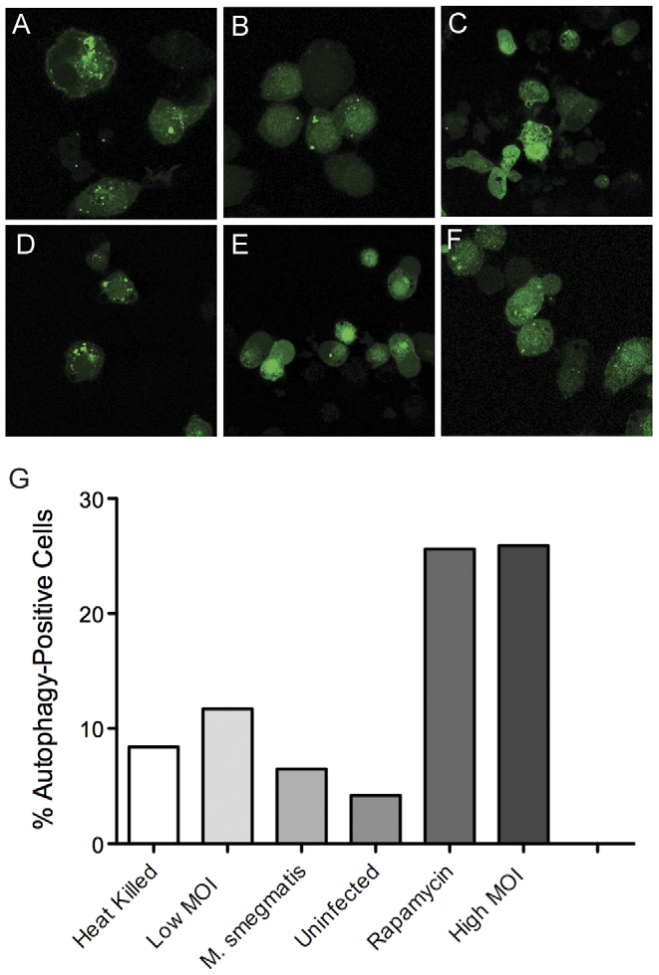
High intracellular burden of *M.tb* induces LC3 aggregation. GFP-LC3 expressing macrophages were infected with bacteria for 6 h as indicated below or treated with rapamycin before being fixed and examined by confocal microscopy for aggregation of LC3 indicative of autophagy. Conditions included (**A**) viable *M.tb* Erdman (MOI 50), (**B**) viable *M.tb* Erdman (MOI 10), (**C**) viable *M. smegmatis* (MOI 50), (**D**) rapamycin (25 µg/ml, 3 h), (**E**) heat-killed *M.tb* Erdman (MOI 50), and (**F**) no infection. Viable *M.tb* Edrman at MOI 50 induced LC3 aggregation as strongly as the positive control, rapamycin. Viable *M.tb* Edrman at MOI 10, viable *M. smegmatis* and heat-killed *M.tb* Erdman did not increase LC3 aggregation compared above the level in uninfected macrophages (**F**). (**G**) The induction of autophagy was quantified by counting the % cells in each group with a number of LC3 aggregates above the median basal level of 5, found in untreated, uninfected macrophages.

In light of our data showing the involvement of iNOS in co-culture conditions, we postulated in macrophages proceeding towards death with a high intracellular burden of *M.tb* bacilli might be delivered to a hostile lysosomal compartment within the cell. Much recent interest has focused on autophagy as a means for macrophages to overcome to arrest of phagosome maturation implemented by *M.tb*. In prior reports, autophagy in *M.tb*-infected macrophages was activated by stimulation with exogenous rapamycin, interferon-γ or ATP [17]. We speculated that bacilli in heavily infected macrophages were being delivered to autophagosomes despite the absence of exogenous autophagy-inducing factors. To test that possibility we generated a mouse macrophage cell line with stable expression of the autophagosome marker LC3 fused to GFP [18]. In the absence of infection uninfected, green fluorescence was homogenously distributed throughout the cytosol of GFP-LC3 macrophages with rare foci of aggregation presumably reflecting an expected basal level of autophagosome formation (Fig. 6). Induction of autophagy with rapamycin as a positive control was reflected by a significant increase in GFP-LC3 aggregation. A similarly strong induction of GFP-LC3 aggregation was observed in macrophages infected with viable *M.tb* at an MOI (50) sufficient to initiate cell death and the antimycobacterial response in co-culture with naïve macrophages. In contrast, GFP-LC3 aggregation was not induced by heat-killed *M.tb* at MOI 50 or by viable *M.tb* at MOI 5; both conditions previously shown not to result in macrophage cell death [4], [19]. The specificity of autophagy induction by viable *M.tb* at high MOI was demonstrated by the failure of viable *E. coli* to stimulate GFP-LC3 aggregation at MOI 50. These results demonstrated that autophagy was specifically induced by a high intracellular burden of *M.tb* in the absence of IFN-γ or other known exogenous factors.

**Figure 7:**
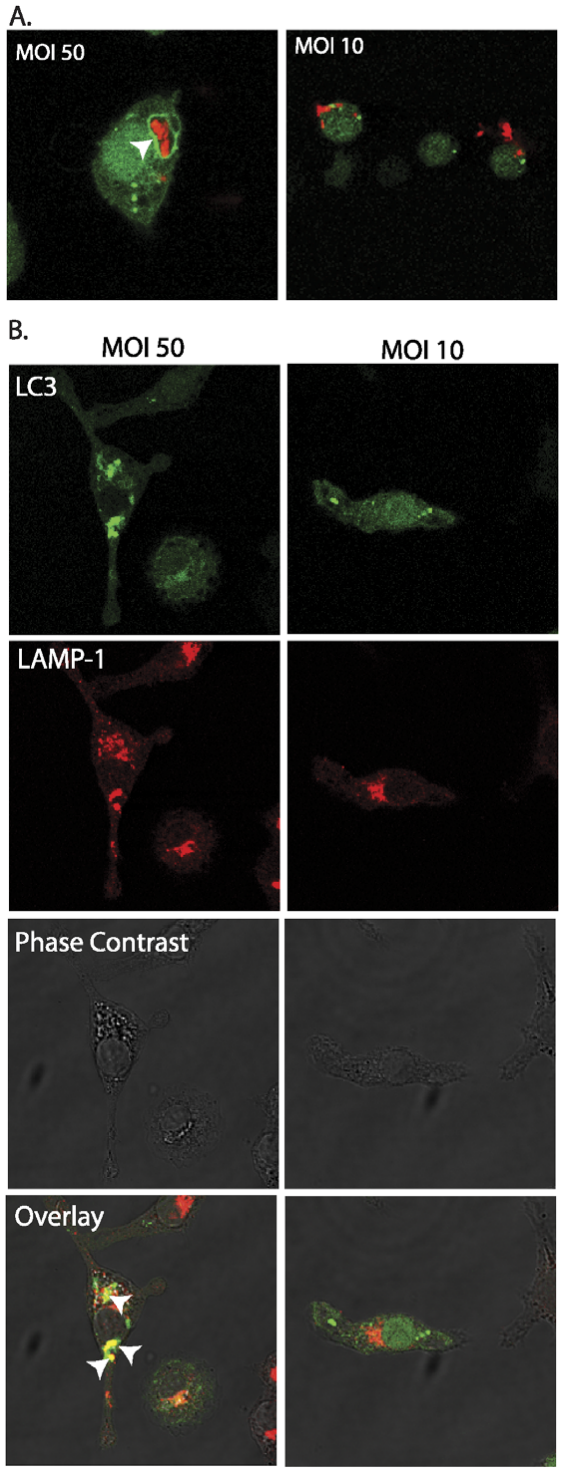
*M.tb* localizes to LC3-positive compartments. (**A**) GFP-LC3 expressing macrophages were infected with mCherry-*M.tb* (MOI 50 or 10, 6 h) and then fixed and observed by confocal microscopy. At high MOI there are GFP-LC3 rings surrounding (*arrowhead*) around mCherry-*M.tb* and co-localization indicated by yellow pixels (*arrow*) that are not seen in macrophages challenged at low MOI. (**B**) LAMP1 immunostaining of GFP-LC3 cell line macrophages infected with *M.tb* Edrman (MOI 50 or 10, 5 h) demonstrates co-localization of LC3 and lysosomal markers (*arrowheads*).

To confirm that autophagy in heavily infected macrophages delivered bacilli to autophagosomes, macrophages were infected with red-fluorescent mCherry-*M.tb* and examined by confocal microscopy. As shown in Figure 7A, mCherry-*M.tb* was located in vacuoles with circumferential green GFP-LC3 fluorescence. Co-localization of mCherry-*M.tb* and GFP-LC3 was quantified in confocal images of macrophages challenged at low vs. high MOI, confirming a ∼60% higher frequency in the high MOI macrophage cultures (Fig. S3).

The final stage of phagosome maturation is typically characterized by loss of late endosomal markers and fusion with lysosomes. LAMP1 is a marker commonly associated with lysosomal fusion. To investigate whether the LC3 positive compartments arising in macrophages with high intracellular burden of *M.tb* were undergoing such maturation, GFP-LC3 expressing cell line macrophages were infected with *M.tb* and subsequently immunostained with anti-LAMP1 antibodies. Under these conditions, co-localization of LC3 and LAMP1 was evident (Fig. 7B). Mature endosomal compartments are also characterized by low pH. In parallel experiments with *M.tb*-infected GFP-LC3 macrophages, we observed co-localization of LC3 with LysoTracker Red DND-99 that partitions to acidified compartments (Fig. S4). Collectively, these results indicate that a high intracellular burden of *M.tb* promotes autophagy in macrophages that delivers bacilli to acidified autolysosomal compartments where nitric oxide-dependent antimycobacterial mechanisms may operate.

## Discussion

Macrophage cell death is thought to play a major role in TB pathogenesis [20]. Previous studies have shown antimicrobial activity when *M.tb-*infected macrophages with features of apoptosis are co-cultured with naïve macrophages [4]. However, when the *M.tb-*infected macrophages are necrotic at the time of co-culture, there is no antimicrobial effect. This suggested that efferocytosis of apoptotic bodies containing *M.tb* might enhance antimicrobial activity of naïve macrophages. However, in vivo studies using *M. marinum* infected zebrafish raised doubt by demonstrating a role for efferocytosis to spread infection to naïve macrophages recruited to granulomas and ultimately to seed distal sites [21]. In the present study we demonstrate that the antimicrobial activity against *M.tb* conferred by naïve macrophages in co-culture depends on a lethal intracellular bacillary burden in the infected macrophages, but efferocytosis is not required.

Transwell cultures demonstrated contact-independent crosstalk where an afferent signal from infected macrophages stimulated an efferent signal from naïve macrophages that restricted *M.tb* replication in the former. The nature of the afferent signal was investigated, with the assumption that cytokines or death-related signals such as nucleotides would be involved. We found an abundance of IL-1β in co-culture supernatants, and additionally, the necessity for IL-1R expression on the naïve macrophage population for antimicrobial activity in co-cultures (Fig. 4). *M.tb*-infected macrophages produced IL-1β and levels were further increased in co-cultures. Giacomini *et al.* [22] showed that *M.tb* infection increases IL-1β production in macrophages and recent studies demonstrated that efferocytosis of *M.tb*-infected apoptotic neutrophils by naïve macrophages up-regulates IL-1β mRNA expression [23]. The importance of IL-1β in our system is inarguable but our experiments did not test whether naïve macrophages also produce IL-1β in co-cultures, nor did they exclude other signals acting synergistically with IL-1β.

Supernatants from co-cultures were capable of stimulating antimicrobial activity in isolated cultures of *M.tb*-infected macrophages. This strongly implied that naïve macrophages in this system produce a soluble factor that augments antimicrobial activity in the infected cell population but an extensive search failed to reveal the identity of this factor. The activity is heat stable, <3 kDa, and resistant to DNase, RNase and proteinase K (Fig. S1a). Previous studies have shown that nucleotide signaling in macrophages stimulated antimycobacterial mechanisms [8], [24] and nucleotides are recognized death-associated molecules. However, our studies failed to identify any involvement of ATP or adenosine signaling. Lipid signaling molecules could fit the physical criteria for the efferent factor in co-culture supernatants, but inhibiting cyclooxygenase with indomethacin had no effect in our system. Our data have so far excluded a wide range of candidates but the identity of the factor that promotes antimycobacterial activity in heavily infected macrophages remains unknown at this time.

While failing to identify the efferent signal in the co-culture system, we were able to gain insight to the antimicrobial mechanisms stimulated by crosstalk between naïve and infected macrophages. Antimicrobial activity is expressed exclusively in the pre-necrotic phase of *M.tb*-induced cell death, when few extracellular bacilli were observed by confocal microscopy. This suggested an antimicrobial mechanism operating within the infected macrophages, a conclusion supported by our finding that the infected cells must express iNOS in order to restrict *M.tb*. This cytoplasmic enzyme normally localizes to phagosomes containing ingested bacteria to efficiently target its product, nitric oxide.

A key feature of *M.tb* virulence is the capacity to prevent its endosomal compartment from progressing through the endocytic pathway [15], [25]. In addition to circumventing exposure to low pH and lysosomal hydrolases, *M.tb* was also shown to inhibit iNOS recruitment to phagosomes [16], [26]. Given the requirement for iNOS, we questioned whether autophagy provided an alternate mechanism to overcome the phagosome biogenesis block by *M.tb*. Autophagy has been described as a defense mechanism against intracellular pathogens including *M.tb* [8], [27]–[29]. In addition to the conventional antimicrobial properties of acidified phagolysosomes, autophagy results in the generation of neo-antimicrobial peptides from processing of ubiquinated cytosolic proteins that are capable of killing *M.tb* [30]. Published studies have so far relied on starvation or exogenous agents such as IFN-γ or rapamycin to induce autophagy in *M.tb*-infected macrophages. We found that *M.tb* infection can directly induce autophagy in macrophages without any requirement for starvation or exogenous factors. This occurs specifically in the context of a lethal intracellular bacillary burden and presumably reflects a stress response in cells committed to death. The specificity of this response to a high intracellular burden of viable *M.tb* was shown by the lack of autophagy induction by heat-killed *M.tb* at high MOI, viable *M.tb* at low MOI, viable *M. smegmatis* at high MOI or viable *E. coli* at high MOI. In the absence of co-culture with naïve macrophages, this autophagic response does not appear to significantly reduce bacillary viability. That may be due a relatively brief time for exposure of bacilli to hostile conditions before membrane-bound compartments the cells disintegrate with progression to necrosis [19]. The soluble factor promoting iNOS-dependent antimicrobial activity in these dying cells could act in any of several ways. It might accelerate delivery of *M.tb* to autolysosomes, it might amplify iNOS enzymatic activity or localization to mycobacterial vacuoles, it might amplify autophagy-specific antimicrobial mechanisms [30], or it might delay macrophage necrosis and thereby prolong the exposure of bacilli to degradative processes within intact autolysosomes.

In conclusion, we studied interactions between *M.tb*-infected and naïve macrophages that model events in pulmonary TB when alveolar macrophages initially infected by inhaled *M.tb* become surrounded by an excess of recruited, naïve macrophages and before the expression of adaptive immunity that restricts bacillary replication [31]. In this early, innate phase of the host response to *M.tb* relatively unrestricted replication leads to high intracellular bacillary loads in vivo as we recently showed [19]. Virulent *M.tb* inhibits macrophage apoptosis but ultimately triggers necrosis at high bacillary loads, making it unlikely that antimicrobial mechanisms dependent on efferocytosis play a major role in TB defense. Our data reveal a 2-way interaction where *M.tb*-infected macrophages committed to cell death produce a signal that causes naïve macrophages to respond by producing a factor which promotes iNOS-dependent inhibition of *M.tb* replication in autolysosomes. The afferent signal requires IL-1β but additional co-factors may be involved. The identity of the efferent signal is presently unknown but our data permit a focused search by excluding DNA, RNA, proteins and molecules >3 kDa. The co-culture antimycobacterial effect we describe here may represent a previously unrecognized innate immunological defense mechanism that may help limit the maximal replicative potential of *M.tb* prior to the expression of adaptive immunity in the newly infected host.

## Materials and Methods

### Ethics statement

Experiments with animals were conducted according to the National Institutes of Health guidelines for housing and care of laboratory animals under protocols approved by the Institutional Animal Care and Use Committee (A-1429) and the Institutional Biosafety Committee (I-161) at The University of Massachusetts Medical School.

### Reagents

Autophagy inhibitor 3-methyladenine, the non-selective P2X_7_ receptor agonist 5′-N-ethylcarboxamidoadenosine (NECA), ribonucelase A1, proteinase K (from *Tritirachium album*), cyclooxygenase inhibitor indomethacin and polyclonal rabbit-anti mouse LAMP1 antibodies were purchased from Sigma Aldrich. The mTOR inhibitor rapamycin was purchased from Cell Signaling. TURBO DNase was purchased from Ambion. Centrifugal filter devices used for size fractionation based on Stoke's radius were purchased from Millipore. Secondary antibody Alexa Fluor 647 goat-anti-rabbit IgG detection for LAMP1 immunofluorescence was purchased from Molecular Probes.

### Cell culture and mice

Murine macrophage cell lines were made from C57/BL6 wildtype mice, iNOS^-/-^ mice (Jackson Labs), IL-1R^-/-^ mice (gift of Dr. K. Fitzgerald, University of Massachusetts Medical School) using a J2 transforming retrovirus previously described. [32], [33] Bone marrow from P2X_7_R^-/-^ mice were provided by Dr. R. Ingalls (Boston University School of Medicine) and Dr. A. Hise (Case Western Revere University), and A2a R^-/-^ mice were kindly provided by Dr. J.F. Chen (Boston University School of Medicine). Creation of the GFP-LC3 vector and macrophages are described by Harris et al. [18]. Unless otherwise stated, macrophages used in co-culture experiments were cell lines. Cell lines were maintained in DMEM medium (Invitrogen Life Technologies) supplemented with 10% FBS (BioWhittaker), 100 U/mL penicillin, 100 mg/mL streptomycin and 2 mM glutamine and 10% L-929 conditioned media, prepared in-house, is added for culturing bone marrow derived macrophages.

### Bacterial strains and infections

*M.tb* Erdman, used for all experiments unless otherwise indicated was obtained from Trudeau Institute. *M. smegmatis* mc^2^155 was purchased from ATCC. mCherry-*M.tb*, a generous gift from Dr. C. Sassetti (University of Massachusetts Medical School), was synthesized and codon-optimized with the mCherry open reading frame cloned into a pAL5000 based multicopy plasmid under the control of an optimized *E. coli* promoter in the *M.tb* H37Rv background. Bacteria were cultured in roller bottles containing Middlebrook 7H9 broth to OD_600_ of 0.7, washed twice in PBS and resuspended in DMEM medium supplemented with 10% FBS and 2 mM glutamine at 1.5 − 2×10^8^ bacteria per mL. Stocks of bacteria were frozen immediately at -80°C. Heat killed *M.tb* was generated by boiling bacteria at 100°C for 30 min.

To infect cells, an aliquot of *M.tb* culture was thawed and dispersed with water bath sonicator (Branson Ultrasonics) for 90 sec at room temperature. Cultures were allowed to settle for 20 min to remove clumped bacteria and then macrophages were then infected at MOI 10 or 50 for 3 h at 37°C, 5% CO_2_. Infected macrophages were washed twice with DMEM and incubated for an additional 1 h.

CFU was measured at 0, 24, 48 or 72 h after the addition of the naïve macrophages by incubating the cells in 0.5 mL 0.05% Tween 20 in water for 5 min at room temperature. Cultures were agitated by pipetting and serial diluted in 1 mL aliquots of 0.05% Tween 80 in PBS then plated onto Middlebrook 7H11 agar plates. Plates were incubated at 37°C until colonies were counted 16 and 21 days after plating.

### Co-culture experiments

2×10^5^ macrophages were incubated for 1 h in 24-well polystyrene tissue culture plates (Nalge Nunc International) and then were infected as described above. At 4 h post-infection, an equal number of naïve macrophages were added either directly to wells containing infected macrophages, or to 0.4 µm pore-size, polycarbonate transwell inserts (Corning Inc.). Co-cultures were incubated for various time points before lysis of infected cells and plating for CFU. In supernatant transfer experiments, supernatants were collected from co-cultures of naïve and *M.tb*-infected macrophages 3 h after the naïve cells were added. Supernants were filter-sterilized (0.22 µm) and then added to macrophages that had been infected as described above.

### Multiplex

Supernatants from macrophages infected at MOI 50 and supernatants from high MOI co-cultures were collected at 3 h post-addition of naïve macrophages or 7 h post-infection. Supernatants were passed through a 0.22 µm pore size syringe filter then frozen at −80°C until shipped for multiplex analysis (Aushon Biosystems).

### Macrophage staining and fluorescent laser scanning confocal microscopy

Immortalized cell line macrophages were stained with cholera toxin subunit B conjugates 488 or 647 (Molecular Probes) at 4°C for 30 min and then washed three times with DMEM. For confocal microscopy, cells were then plated at 10^6^ cells per 35 mm glass bottom plate with 10 mm glass No. 1.5 thickness (MatTek). After infection and/or co-culturing, contents of plates were fixed with 4% paraformaldehyde in PBS for 30 min then washed three times with PBS.

For LAMP1 immunostaining, GFP-LC3 expressing cell line macrophages were infected at MOI 50 for 3 h then washed twice with DMEM and incubated 2 h further. Cells were fixed with 4% paraformaldehyde in PBS for 30 min then permeabilized with 0.1% Triton X-100 in PBS for 10 min and blocked in blocking buffer (10% goat serum in PBS with 1%BSA and 0.1%Triton X-100; 1 h). Cells were then stained 1:200 anti-LAMP1 antibodies for 1 h in blocking buffer. After washing 5x with PBS, cells were stained with anti-Rabbit secondary antibody conjugated to Alexa Fluor 647 for 1 h. Cells were then washed 5x with PBS. Cells were imaged using a 63x objective at room temperature with a SP2 AOBS confocal laser scanning microscope running LCS software (Leica Microsystems).

### Quantifying cell-cell contact

After confocal imaging, broad fields of cells were assessed for interactions indicative of efferocytosis. While complete engulfment of infected macrophages by uninfected macrophages was rarely observed, the images revealed frequent cell-cell interactions with a distinct directionality of cell contact having the appearance of frustrated efferocytosis. To quantify the direction of these contacts a “positive” efferocytosis interaction was defined as pedestal formation or incomplete engulfment of one cell by another. A “negative” interaction was one where two cells in contact showed no synapse or pedestal formation and no extension of membrane from one cell partially surrounding another. Results are expressed as % positive interactions vs. % negative interactions.

### Statistics

Differences between groups at the same time-point were assessed by unpaired t-test using GraphPad Prism. Values of *p*≤0.05 were considered to be statistically significant.

## Supporting Information

Figure S1 **Nucleotide signaling does not contribute to the antimycobacterial activity in macrophage co-cultures.** **(A)** Co-cultures were established using primary, bone marrow-derived type macrophages infected with *M.tb* and naïve bone marrow-derived P2X_7_ receptor-deficient macrophages (*WT AB P2X7R−/−*) or vice versa. CFU per mL was measured at the indicated times. The co-culture antimicrobial effect was observed in both conditions. **(B)** Co-cultures were established with *M.tb*-infected wildtype primary macrophages and naïve primary adenosine A2a receptor-deficient macrophages (*WT AB A2a−/−*) and vice versa. CFU per mL was measured at time zero and 48 h post-addition of treated supernatants. Adenosine A2a receptor expression on either cell population was dispensable for *M.tb* growth inhibition.
(TIF)

Figure S2 **Soluble antimicrobial activity in macrophage co-cultures is not mediated by DNA, RNA or protein.** Supernatants were harvested from co-cultures of *M.tb*-infected and naïve macrophages, filter-sterilized and then treated as described before being added to isolated cultures of macrophages infected with *M.tb* (MOI 50, 3 h). CFU per mL was measured at time zero and at the indicated times post-addition of treated supernatants. **(A)** Treating co-culture conditioned supernatant with DNase or RNase failed to eliminate antimicrobial activity. **(B)** Co-culture conditioned supernatants were treated with proteinase K and then heated to 95^o^C for 5 min, or passed through the indicated size-exclusion filters before being added to *M.tb*-infected macrophages. These treatments failed inhibit the ability conditioned supernatant to promote inhibition of *M.tb* replication in the heavily-infected macrophages.
(TIF)

Figure S3 **Co-localization of** ***M.tb*** **with LC3 in infected macrophages.** GFP-LC3 expressing macrophages were infected with mCherry-*M.tb* (MOI 50 or 10, 5 h) and then fixed and observed by confocal microscopy. Cells in the MOI 50 (*high MOI*) and MOI 10 (*low MOI*) groups where *M.tb* was seen to co-localize with LC3 aggregates (see Fig. 7A) were counted. Results are expressed at the % cells with co-localization of the total number of cells counted. ****, *p*<0.0001 comparing co-localization between high and low MOI infected groups.
(TIFF)

Figure S4 ***M.tb*** **localizes to acidified LC3-positive compartments.** GFP-LC3 expressing macrophages were infected with mCherry-*M.tb* at MOI 25 or 10 for 2 h then washed twice with DMEM and incubated 4 h further. 50nM LysoTracker Red DND-99 (Invitrogen) was added to infected macrophages for 20 min at room temperature. Cultures were then washed three times with PBS and fixed with 4% paraformaldehyde in PBS for 30 min. Fixed cells were washed three times with PBS and examined by confocal microscopy. **(A)** At high MOI there are GFP-LC3 rings surrounding (*arrowhead*) around mCherry-*M.tb* and co-localization indicated by yellow pixels (*arrow*) that are not seen in macrophages challenged at low MOI. **(B)** LysoTracker Red DND-99 staining of GFP-LC3 macrophages infected with *M.tb* Edrman (MOI 25, 5 h) demonstrates co-localization of LC3 and LysoTracker (*arrow*).
(TIF)
