# Supplementary figures and images for: Interactions between Naïve and Infected Macrophages Reduce Mycobacterium tuberculosis Viability
Source: PLoS One. 2011 Nov 18;6(11):e27972. doi: 10.1371/journal.pone.0027972 (PMC3220711; doi:10.1371/journal.pone.0027972)

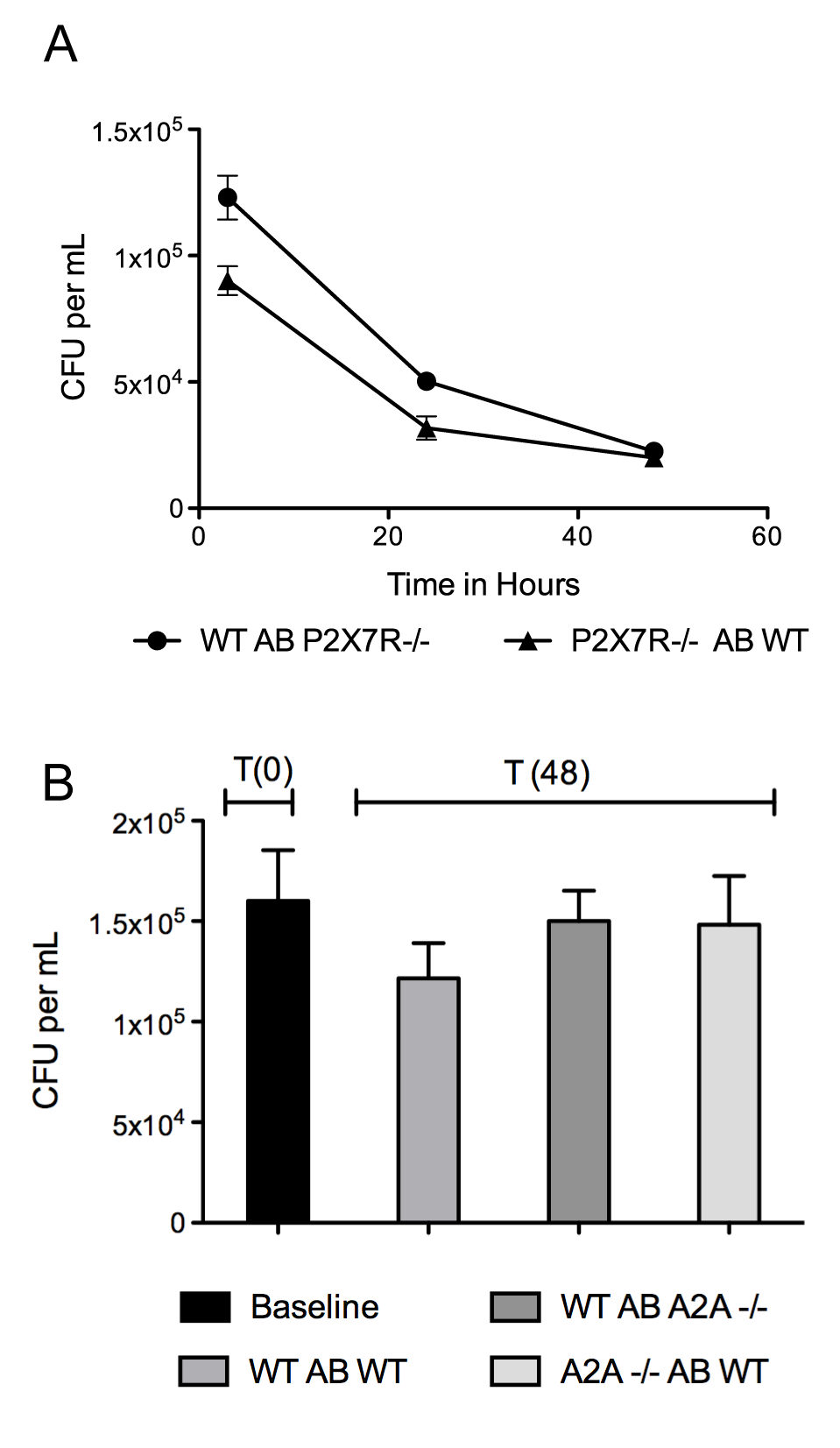

Supplement: Figure S1 — Nucleotide signaling does not contribute to the antimycobacterial activity in macrophage co-cultures. (A) Co-cultures were established using primary, bone marrow-derived type macrophages infected with M.tb and naïve bone marrow-derived P2X7 receptor-deficient macrophages (WT AB P2X7R−/−) or vice versa. CFU per mL was measured at the indicated times. The co-culture antimicrobial effect was observed in both conditions. (B) Co-cultures were established with M.tb-infected wildtype primary macrophages and naïve primary adenosine A2a receptor-deficient macrophages (WT AB A2a−/−) and vice versa. CFU per mL was measured at time zero and 48 h post-addition of treated supernatants. Adenosine A2a receptor expression on either cell population was dispensable for M.tb growth inhibition. (TIF) [file pone.0027972.s001.tif]

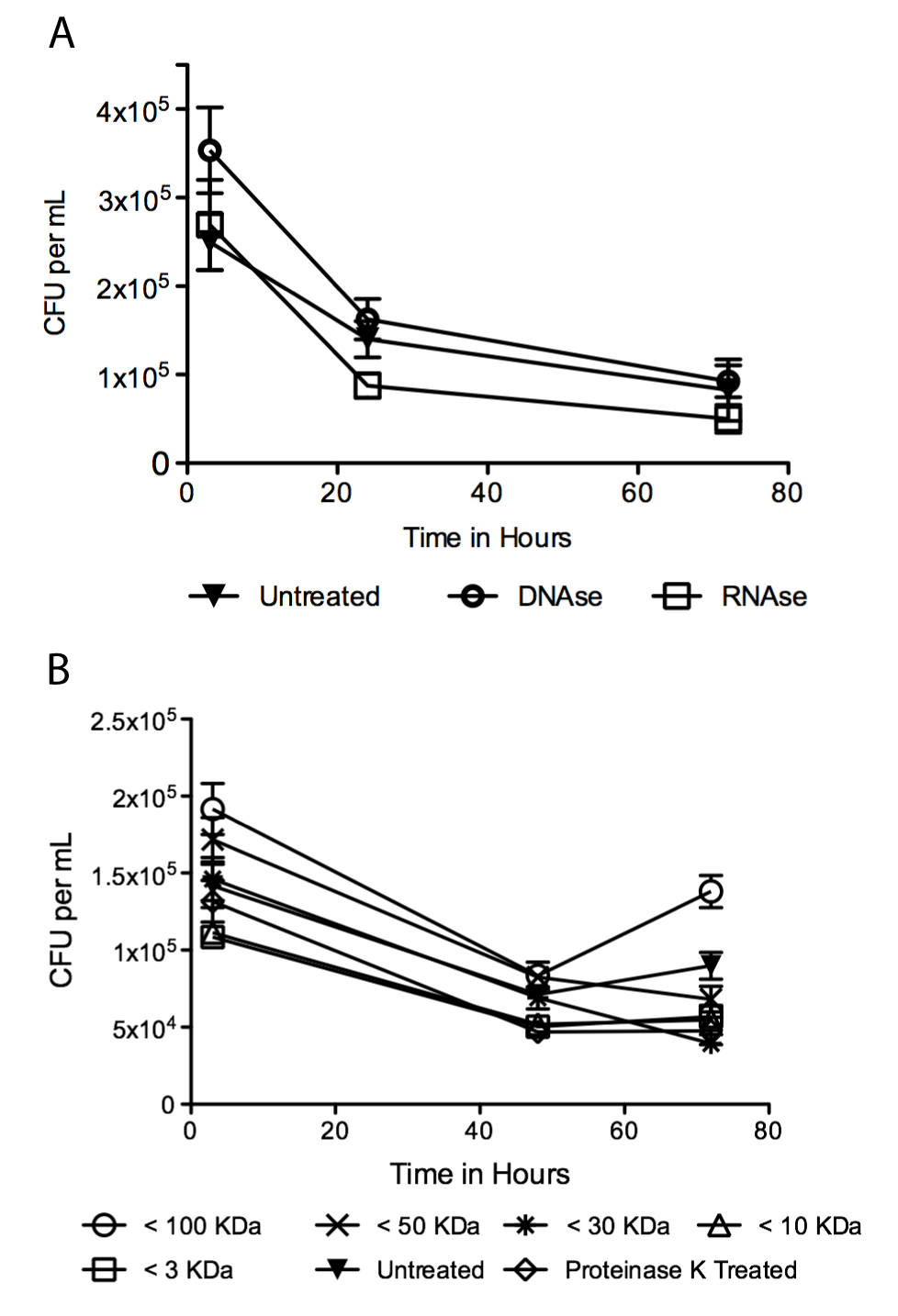

Supplement: Figure S2 — Soluble antimicrobial activity in macrophage co-cultures is not mediated by DNA, RNA or protein. Supernatants were harvested from co-cultures of M.tb-infected and naïve macrophages, filter-sterilized and then treated as described before being added to isolated cultures of macrophages infected with M.tb (MOI 50, 3 h). CFU per mL was measured at time zero and at the indicated times post-addition of treated supernatants. (A) Treating co-culture conditioned supernatant with DNase or RNase failed to eliminate antimicrobial activity. (B) Co-culture conditioned supernatants were treated with proteinase K and then heated to 95oC for 5 min, or passed through the indicated size-exclusion filters before being added to M.tb-infected macrophages. These treatments failed inhibit the ability conditioned supernatant to promote inhibition of M.tb replication in the heavily-infected macrophages. (TIF) [file pone.0027972.s002.tif]

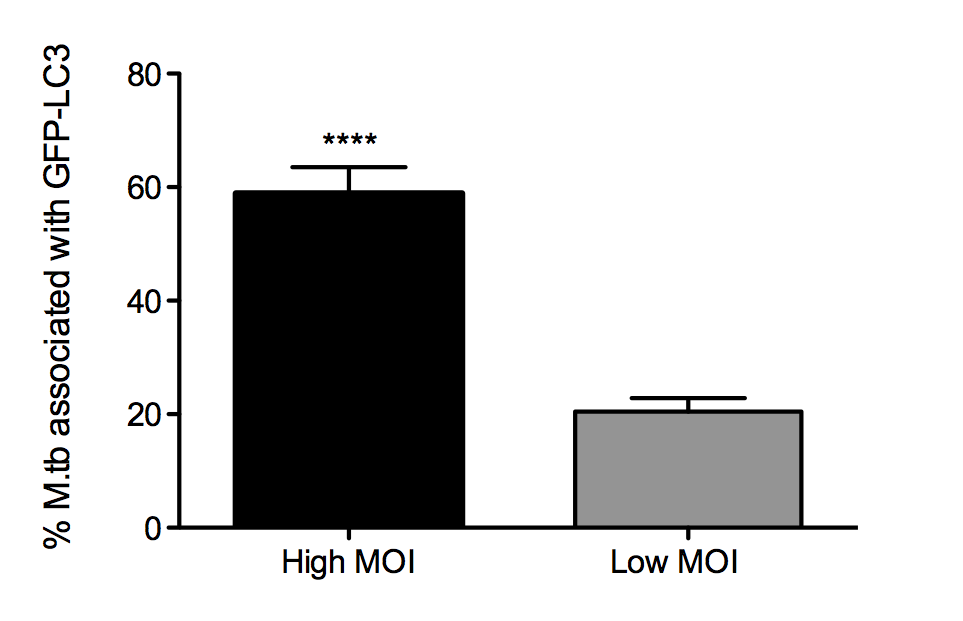

Supplement: Figure S3 — Co-localization of M.tb with LC3 in infected macrophages. GFP-LC3 expressing macrophages were infected with mCherry-M.tb (MOI 50 or 10, 5 h) and then fixed and observed by confocal microscopy. Cells in the MOI 50 (high MOI) and MOI 10 (low MOI) groups where M.tb was seen to co-localize with LC3 aggregates (see Fig. 7A) were counted. Results are expressed at the % cells with co-localization of the total number of cells counted. ****, p<0.0001 comparing co-localization between high and low MOI infected groups. (TIFF) [file pone.0027972.s003.tif]

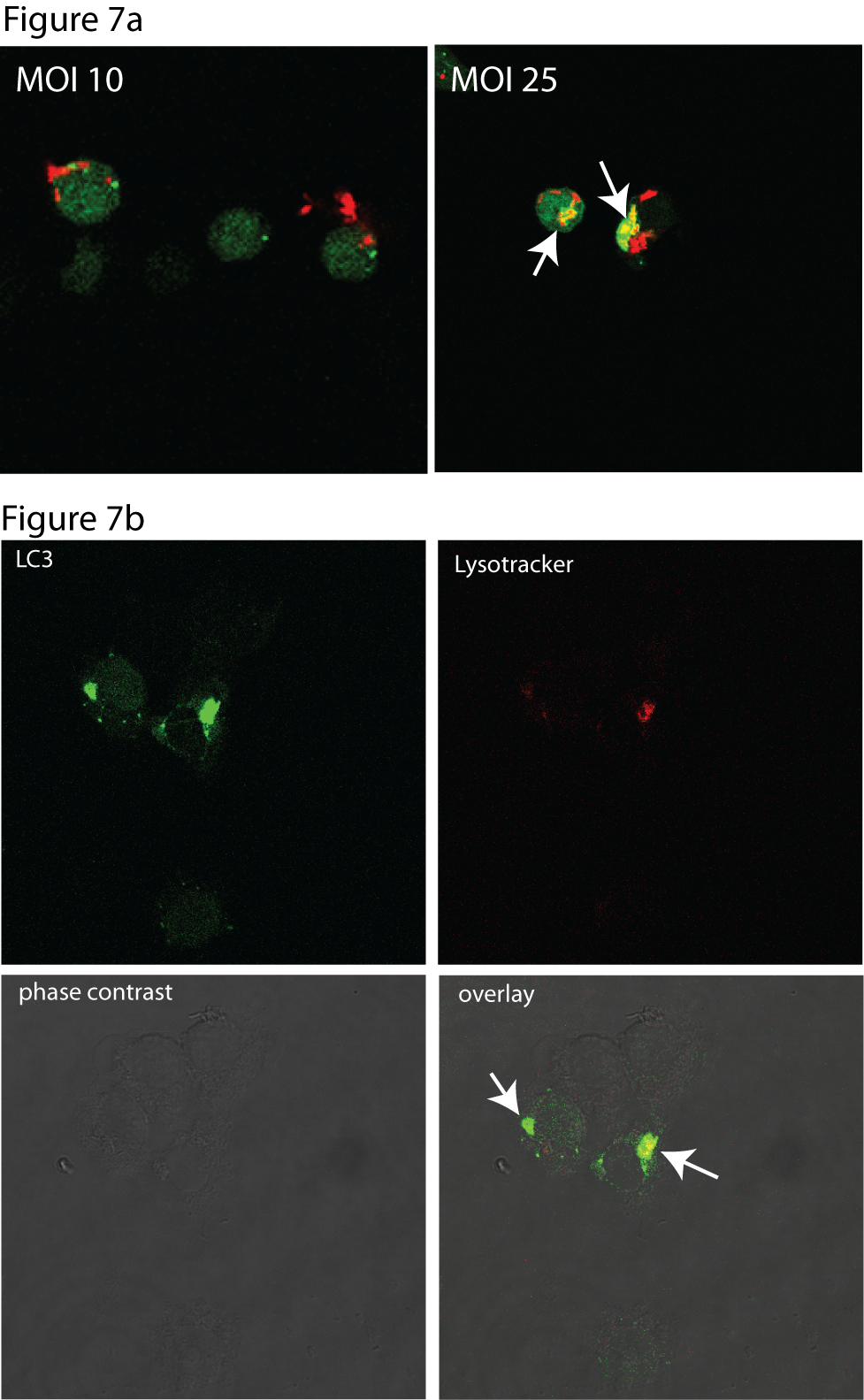

Supplement: Figure S4 — M.tb localizes to acidified LC3-positive compartments. GFP-LC3 expressing macrophages were infected with mCherry-M.tb at MOI 25 or 10 for 2 h then washed twice with DMEM and incubated 4 h further. 50nM LysoTracker Red DND-99 (Invitrogen) was added to infected macrophages for 20 min at room temperature. Cultures were then washed three times with PBS and fixed with 4% paraformaldehyde in PBS for 30 min. Fixed cells were washed three times with PBS and examined by confocal microscopy. (A) At high MOI there are GFP-LC3 rings surrounding (arrowhead) around mCherry-M.tb and co-localization indicated by yellow pixels (arrow) that are not seen in macrophages challenged at low MOI. (B) LysoTracker Red DND-99 staining of GFP-LC3 macrophages infected with M.tb Edrman (MOI 25, 5 h) demonstrates co-localization of LC3 and LysoTracker (arrow). (TIF) [file pone.0027972.s004.tif]
